# Long-Term Effects of Neural Precursor Cell Transplantation on Secondary Injury Processes and Functional Recovery after Severe Cervical Contusion-Compression Spinal Cord Injury

**DOI:** 10.3390/ijms222313106

**Published:** 2021-12-03

**Authors:** Alexander Younsi, Guoli Zheng, Lennart Riemann, Moritz Scherer, Hao Zhang, Mohamed Tail, Maryam Hatami, Thomas Skutella, Andreas Unterberg, Klaus Zweckberger

**Affiliations:** 1 Department of Neurosurgery, University Hospital Heidelberg, University of Heidelberg, INF 400, 69120 Heidelberg, Germany; guo3li4.zheng4@gmail.com (G.Z.); lennart-riemann@posteo.de (L.R.); moritz.scherer@med.uni-heidelberg.de (M.S.); zhanghaoayfy523@gmail.com (H.Z.); tailmohamed@outlook.com (M.T.); andreas.unterberg@med.uni-heidelberg.de (A.U.); K.Zweckberger@klinikum-braunschweig.de (K.Z.); 2 Department of Neuroanatomy, Institute for Anatomy and Cell Biology, University of Heidelberg, INF 307, 69120 Heidelberg, Germany; maryam.hatami@uni-heidelberg.de (M.H.); thomas.skutella@uni-heidelberg.de (T.S.)

**Keywords:** SCI, spinal cord injury, stem cell therapy, neuronal precursor cells, NPCs, neuroregeneration, functional recovery

## Abstract

Cervical spinal cord injury (SCI) remains a devastating event without adequate treatment options despite decades of research. In this context, the usefulness of common preclinical SCI models has been criticized. We, therefore, aimed to use a clinically relevant animal model of severe cervical SCI to assess the long-term effects of neural precursor cell (NPC) transplantation on secondary injury processes and functional recovery. To this end, we performed a clip contusion-compression injury at the C6 level in 40 female Wistar rats and a sham surgery in 10 female Wistar rats. NPCs, isolated from the subventricular zone of green fluorescent protein (GFP) expressing transgenic rat embryos, were transplanted ten days after the injury. Functional recovery was assessed weekly, and FluoroGold (FG) retrograde fiber-labeling, as well as manganese-enhanced magnetic resonance imaging (MEMRI), were performed prior to the sacrifice of the animals eight weeks after SCI. After cryosectioning of the spinal cords, immunofluorescence staining was conducted. Results were compared between the treatment groups (NPC, Vehicle, Sham) and statistically analyzed (*p* < 0.05 was considered significant). Despite the severity of the injury, leading to substantial morbidity and mortality during the experiment, long-term survival of the engrafted NPCs with a predominant differentiation into oligodendrocytes could be observed after eight weeks. While myelination of the injured spinal cord was not significantly improved, NPC treated animals showed a significant increase of intact perilesional motor neurons and preserved spinal tracts compared to untreated Vehicle animals. These findings were associated with enhanced preservation of intact spinal cord tissue. However, reactive astrogliosis and inflammation where not significantly reduced by the NPC-treatment. While differences in the Basso–Beattie–Bresnahan (BBB) score and the Gridwalk test remained insignificant, animals in the NPC group performed significantly better in the more objective CatWalk XT gait analysis, suggesting some beneficial effects of the engrafted NPCs on the functional recovery after severe cervical SCI.

## 1. Introduction

Spinal cord injury (SCI) remains a devastating event leading to loss of independence and often lifelong disability while incurring high socioeconomic costs [1,2]. However, despite decades of research, no neuroprotective or neuroregenerative treatment has been successfully translated into the clinical setting, and existing surgical as well as medical therapies, are limited. While the cervical spine is involved in more than 60% of patients with SCI, affecting the lower but also the upper extremities and often impairing bladder as well as bowel function, trunk stability, and even respiration, most preclinical studies have been performed in the thoracic spine [3,4]. Moreover, sharp and unilateral SCI models (e.g., hemisection) have predominantly been used, typically resulting in only limited secondary injury processes [5]. In contrast, primary damage to the spinal cord in clinical SCI usually occurs due to contusion and compression, leading to subsequent secondary injury cascades with further loss of neural tissue, demyelination, and neuroinflammation [6]. In the emerging hostile microenvironment, endogenous regeneration, mediated by progenitor populations that are capable of replacing lost tissue, is additionally impaired by the development of astroglial/proteoglycan scars and cystic cavities that form physical and chemical barriers to axonal outgrowth and cell migration [7,8,9]. The transplantation of exogenous stem cells into the injured spinal cord to mimic such repair processes has thus become increasingly popular in recent years [10,11] and has shown promising results by, e.g., inhibiting inflammatory signaling, releasing trophic factors, or supplying an extracellular matrix [12,13]. In this context, neural precursors cells (NPCs) have been especially attractive because they differentiate into both neurons and glial cells promoting remyelination and improving functional recovery after transplantation while not forming tumors in existing preclinical studies [8,14,15,16,17]. Nevertheless, clinical trials using transplantation of NPCs to treat SCI had limited success so far, indicating that the models used to generate preclinical data might not have been suitable to warrant clinical translation [18,19]. As proposed by a growing number of researchers, the efficacy of preclinical studies should, therefore, be increased by, e.g., using more clinically relevant SCI models or better understanding the connection between specific histological changes and functional recovery [20,21]. In our current study, we, therefore, used a contusion-compression model of cervical SCI to assess the long-term effects of NPC-transplantation on relevant tissue and neurobehavioral outcomes, including myelination, spinal tract, and spinal cord tissue preservation, astroglial scarring, inflammation as well as gait and front- and hindlimb function.

## 2. Results

### 2.1. Survival and Differentiation of NPCs

**Figure 1:**
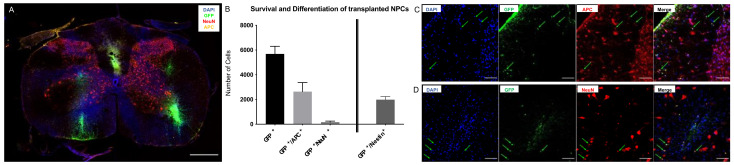
Survival and differentiation of transplanted NPCs eight weeks after severe cervical SCI. (**A**) Representative image of an NPC animal spinal cord cross-section at 10× magnification depicting the spatial distribution of transplanted NPCs (GFP^+^/DAPI^+^), NPC-derived neurons (GFP^+^/DAPI^+^/NeuN^+^) and NPC-derived oligodendrocytes (GFP^+^/DAPI^+^/APC^+^; scale bar = 500 µm). (**B**) Mean number of surviving NPCs (GFP^+^), NPC-derived mature neurons (GFP^+^/NeuN^+^), NPC-derived mature oligodendrocytes (GFP^+^/APC^+^) and undifferentiated NPCs (GFP^+^/Nestin^+^) in the injured spinal cord of animals in the NPC group (group 1; *n* = 8 animals). (**C**) Colocalization (green arrows) of DAPI, GFP and APC, indicating surviving, viable and mature NPC-derived oligodendrocytes and of (**D**) DAPI, GFP and NeuN, indicating surviving, viable and mature NPC-derived neurons in an NPC animal at 40× magnification (scale bar = 100 µm).

Long-term survival of the cell-graft is an important indicator of cell transplantation efficacy. In our study of cervical clip contusion-compression SCI with intraspinal transplantation of the NPCs in the subacute phase, engrafted cells were verifiable in the spinal cord even in the chronic phase eight weeks after the injury. Quantification of GFP^+^/DAPI^+^ cells in NPC animals (group 1) revealed 5689 ± 612 viable NPCs (Figure 1A,B) with a rostro-caudal distribution over a length of 4.1 ± 0.35 mm, suggesting an in- as well as outward migration from the transplant zone with a typical location of the transplanted cells in the dorsal white or gray matter. Most of the surviving NPCs had differentiated towards the oligodendroglial lineage with 2636 ± 732 cells expressing GFP and the adenomatous polyposis coli (APC) protein, a marker for mature oligodendrocytes (46% of the surviving NPCs with a GFP^+^/APC^+^ mature oligodendroglial immunophenotype; Figure 1B,C). Differentiation towards the neuronal lineage was substantially lower, with 172 ± 74 cells expressing GFP and the neuronal nuclear antigen (NeuN), a marker for mature neurons (3% of the surviving NPCs with a GFP^+^/NeuN^+^ mature neuronal immunophenotype; Figure 1B,D). Eight weeks after SCI, a substantial part of the surviving cell graft (1987 ± 254 cells) was still in undifferentiated state as indicated by the co-expression of GFP and the stem-cell marker Nestin (35% of surviving NPCs with a GFP^+^/Nestin^+^ stem-cell immunophenotype; Figure 1B).

### 2.2. Spared Motor Neurons and Myelination

**Figure 2:**
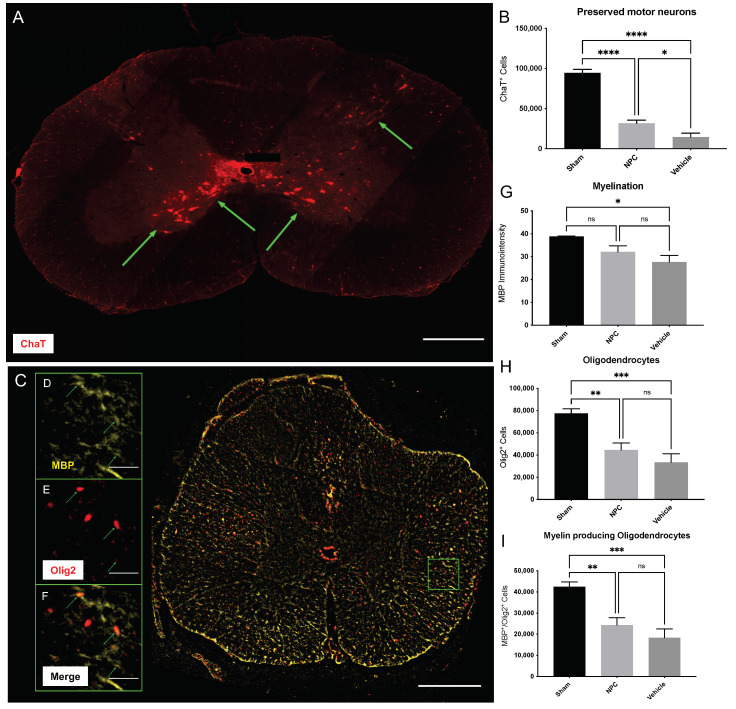
Spared motor neurons, oligodendrocytes, and myelination in the injured spinal cord eight weeks after severe cervical SCI. (**A**) Spinal cord cross-section of an NPC animal stained for the motor neuron marker ChaT, indicating spared motor neurons (green arrows) in the ventral grey matter at 10× magnification (scale bar = 500 µm). (**B**) Significantly more ChaT^+^ motor neurons could be observed in NPC animals (group 1) compared to Vehicle animals (group 2; *n* = 8 animals per group; one-way analysis of variance (ANOVA) with post-hoc Tukey-HSD-test; *p* = 0.0336). (**C**) Spinal cord cross-section of a Vehicle animal stained for the oligendroglial marker Olig2 and the surrogate marker for myelin MBP at 10× magnification (scale bar = 500 µm). (**D**–**F**) Enlargement of the green framed inlet in (**C**), depicting the close spatial relationship (green arrows) of active Olig2^+^ oligodendrocytes and MBP at 40× magnification (scale bar = 50 µm). (**G**) The immuno-intensity of the MBP-staining and thus the extent of myelination showed no significant difference between NPC animals and Vehicle animals (*n* = 8 animals per group; one-way ANOVA with post-hoc Tukey-HSD-test; *p* = 0.3945). Similarly, the number of Olig2^+^ oligodendrocytes (**H**) and Olig2^+^/MBP^+^ active oligodendrocytes (**I**) were not significantly higher in NPC animals compared to Vehicle animals (both *n* = 8 animals per group; one-way ANOVA with post-hoc Tukey-HSD-test; *p* = 04549) (* *p* < 0.05; ** *p* < 0.01; *** *p* < 0.001; **** *p* < 0.0001 and ns = not significant).

As the preservation and regeneration of motor neurons might be closely related to functional recovery after SCI, we used the motor neuron marker choline acetyltransferase (ChaT) to assess the number of spared or regenerated perilesional motor neurons eight weeks after the injury (Figure 2A). Quantitative analysis of ChaT^+^ cells, typically located in the ventral horns of the grey matter, showed significantly more perilesional motor neurons in NPC animals (group 1) compared to Vehicle animals (group 2; 31,840 ± 3842 vs. 14,613 ± 4830 Chat^+^ cells; *p* = 0.0336; Figure 2B). At the same time, uninjured Sham animals (group 3) displayed a significantly higher number of motor neurons (94,366 ± 4551 Chat^+^ cells) compared to both, NPC (*p* < 0.0001) and Vehicle animals (*p* < 0.0001).

We, therefore, concluded that the transplantation of NPCs in the subacute phase significantly improves the preservation or regeneration of motor neurons in the chronic phase after cervical SCI.

Chronic progressive demyelination is considered to be a significant contributor to the functional deficits occurring after SCI [22]. By quantifying the immuno-intensity of myelin basic protein (MBP), which is expressed by oligodendrocytes to maintain the correct structure of myelin, the degree of myelination in the spinal cord eight weeks after the injury was assessed (Figure 2C). In addition, cells expressing the oligodendrocyte transcription factor (Olig2), a marker for oligodendrocytes, were visualized alone (Olig2^+^) and with a close spatial relationship to MBP (Olig2^+^/MBP^+^), to identify the overall number of oligodendrocytes and MBP-producing, functional oligodendrocytes in the spinal cord tissue (Figure 2D–F). In comparison to the Sham group (group 3), Vehicle animals (group 2) had a significantly lower MBP-immuno-intensity (38.88 ± 0.15 pi/mm^2^ vs. 27.65 ± 2.87 pi/mm^2^; *p* = 0.0155) while the difference to NPC animals (group 1) remained unsignificant (32.1 ± 2.7 pi/mm^2^; *p* = 0.1610). However, no significant difference in the MBP-immuno-intensity could be observed between Vehicle and NPC animals as well (*p* = 0.3945); Figure 2G). While the mean overall number of Olig2^+^ oligodendrocytes per NPC animal was slightly higher in NPC animals (44386 ± 6352 Olig2^+^ cells) compared to Vehicle animals (33484 ± 7604 Olig2^+^ cells), this difference remained unsignificant as well (*p* = 0.4549). Only animals in the Sham group showed significantly more oligodendrocytes (77,477 ± 4157 Olig2^+^ cells) compared to both, NPC (*p* = 0.0092) and Vehicle animals (*p* = 0.0009; Figure 2H). Similarly, the number of MBP^+^/Olig2^+^ active oligodendrocytes was higher in the NPC group compared to the Vehicle group (24,320 ± 3480 vs. 18,347 ± 4166 MBP^+^/Olig2^+^ cells), but this difference did not reach significance either (*p* = 0.4549; Figure 2I). Animals in the Sham group, on the other hand, displayed significantly more active oligodendrocytes (42,451 ± 2278 MBP^+^/Olig2^+^ cells) than NPC (*p* = 0.0082) and Vehicle animals (*p* = 0.0009; Figure 2I).

These findings led us to the assumption that the transplantation of NPCs is unable to significantly improve the degree of myelination or the number of active oligodendrocytes as well as the overall number of oligodendrocytes eight weeks after severe cervical SCI.

### 2.3. Preservation or Regeneration of Descending and Ascending Spinal Tracts

**Figure 3:**
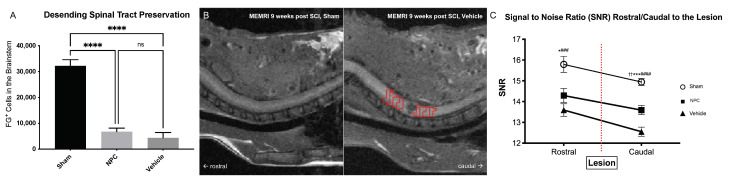
Preservation or regeneration of descending and ascending spinal tracts in the injured spinal cord eight weeks after severe SCI. (**A**) The number of FG^+^ neurons in the brainstem and thus possibly the extent of descending reticulospinal tract preservation or regeneration was not significantly different between NPC animals (group 1) and Vehicle animals (group 2; *n* = 5 animals per group; one-way ANOVA with post-hoc Tukey-HSD-test; *p* = 0.6452). (**B**) On the left side, sagittal T1-weighted MEMRI image of an uninjured Sham animal (group 3) eight weeks after SCI and 48 h after intrathecal injection of MnCl_2_, showing faint but nearly homogeneous contrast enhancement of the cervicothoracic spinal cord. On the right side, sagittal T1-weighted MEMRI image of an injured Vehicle animal (group 2) depicting the lesion as a round, hypointense formation in the cervical spinal cord with suspected caudal decrease of Mn^2+^ signal intensity. Red ROIs depicting the axial slices from 4 mm rostral to 4 mm caudal to the lesion used for detailed SNR measurements. (**C**) Rostral to caudal decrease of SNR over the lesion was only significant in the Vehicle group (*n* = 5 animals, *n* = 4 rostral and *n* = 4 caudal axial slices per animal; unpaired two-sample *t*-test, *p* = 0.007). Correspondingly, SNR caudal to the lesion was significantly lower in Vehicle animals compared to NPC and Sham animals (*n* = 5 animals per group, *n* = 4 caudal axial slices per animal; one-way ANOVA with Tukey-HSD-test; *p* = 0.0017 and *p* < 0.0001, respectively), indicating more preserved or regenerated descending as well as ascending spinal tracts after NPC-transplantation (* *p* < 0.05, NPC vs. Sham; *** *p* < 0.001, NPC vs. Sham; **** *p* < 0.0001, Vehicle vs. Sham and NPC vs. Sham; ### *p* < 0.01, Vehicle vs. Sham; #### *p* < 0.0001, Vehicle vs. Sham; †† *p* < 0.001, NPC vs. Vehicle; ns = not significant).

The recovery of motor and sensory function after SCI is, amongst others, likely dependent upon the preservation or regeneration of descending as well as ascending spinal tracts. We, therefore, analyzed the presence of functional descending tracts eight weeks after SCI by quantification of FG^+^ neurons in the brainstems of animals that had undergone retrograde fiber labeling. With 6760 ± 1369 FG^+^ cells in the whole brainstem, animals in the NPC group (group 1) displayed a slightly higher rate of descending tract preservation than Vehicle animals (group 2; 4368 ± 2072 FG^+^ cells), but this difference was unsignificant (*p* = 0.6452; Figure 3A). Uninjured Sham animals (group 3) on the other hand showed a significantly higher rate of intact descending tracts (32,241 ± 2352 FG^+^ cells) compared to both, the NPC (*p* < 0.0001) and the Vehicle animals (*p* < 0.0001; Figure 3A).

Transplantation of NPCs, therefore, does not lead to significant preservation or regeneration of descending tracts across the posttraumatic lesion in the chronic phase after severe cervical SCI.

In addition, we used manganese-enhanced magnetic resonance imaging (MEMRI) to objectify the perilesional preservation or regeneration of ascending and descending spinal tracts eight weeks after SCI. The overall signal-to-noise ratio (SNR) of the cervical and thoracic spinal cord was significantly increased after intrathecal application and subsequent axonal uptake of Mn^2+^ compared to natively scanned animals (15.79 ± 1.48 vs. 8.22 ± 0.31, *p* < 0.0001). Rostral to the lesion, SNR was highest in Sham animals (group 3; 15.79 ± 0.38; Figure 3B,C) followed by NPC animals (group 1; 14.29 ± 0.34), while animals in the Vehicle group already showed the lowest SNR values (group 2; 13.6 ± 0.3; Figure 3B,C). The differences in SNR were significant between Sham and Vehicle animals (*p* = 0.0002) as well as Sham and NPC animals (*p* = 0.0136) but not between NPC and Vehicle animals (*p* = 0.272). The difference of mean rostral and mean caudal SNR and thus the SNR decrease along the spinal cord and in injured animals also across the lesion was only significant in the Vehicle group (1.04 ± 0.1, *p* = 0.007) and not in the Sham but also not in the NPC group (0.82 ± 0.1, *p* = 0.53 and 0.70 ± 0.14, *p* = 0.09). Therefore, SNR caudal to the lesion did not only show a significant difference between Sham and Vehicle/NPC animals (14.94 ± 0.17 vs. 12.55 ± 0.22/13.59 ± 0.22, *p* < 0.0001 and *p* = 0.0004 respectively; Figure 3C) but in contrast to the findings rostral to the lesion also between NPC and Vehicle animals (*p* = 0.0017).

Those changes of SNR suggest that NPC-transplantation leads to an increased number of axons capable of retrograde Mn^2+^-transportation across the lesioned spinal cord and therefore improves preservation or regeneration of descending as well as ascending spinal tracts after severe cervical SCI.

### 2.4. Astrogliosis and Tissue Preservation

**Figure 4:**
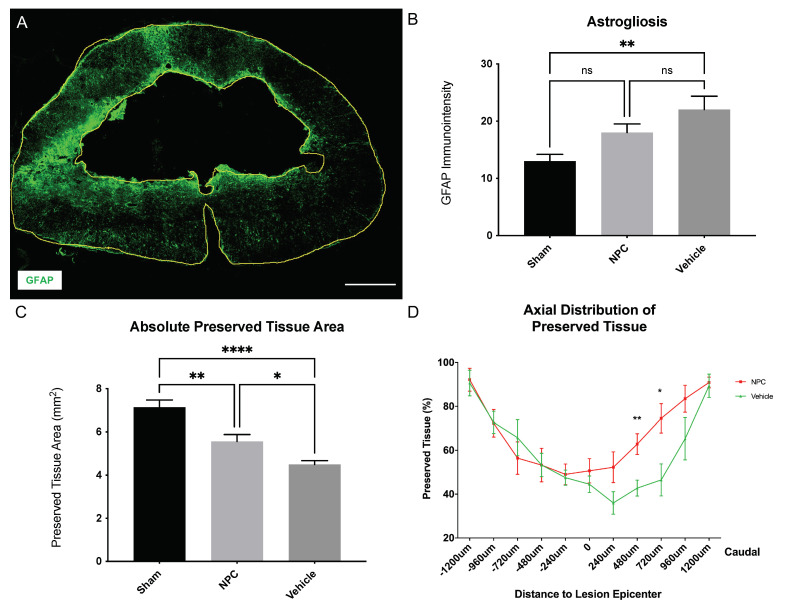
Reactive astrogliosis and tissue preservation in the injured spinal cord eight weeks after severe cervical SCI. (**A**) Spinal cord cross-section of a Vehicle animal stained for GFAP, a marker for reactive astrocytes, at 10× magnification with the entire spinal cord as well as the intramedullary cystic cavity outlined (yellow lines; scale bar = 500 µm). (**B**) The immuno-intensity of the GFAP-staining and thus the reactive astrogliosis was comparable in NPC animals (group 1) and Vehicle animals (group 2; *n* = 8 animals per group; one-way ANOVA with Tukey-HSD-test; *p* = 0.2624). (**C**) The overall preserved tissue area was significantly higher in NPC animals compared to Vehicle animals (*n* = 8 animals per group; one-way ANOVA with Tukey-HSD-test; *p* = 0.043) (**D**) The percentage of preserved spinal cord tissue along the spinal axis was lowest +/−240 µm from the lesion epicenter in all injured animals, with an increase to 90% towards +/−1200 µm. In comparison to Vehicle animals, NPC animals showed a significant increase of preserved tissue 480 µm and 720 µm caudal from the lesion epicenter (*n* = 8 animals per group; one-way ANOVA with Tukey-HSD-test; *p* = 0.0071 and *p* = 0.0177, respectively) (* *p* < 0.05; ** *p* < 0.01; **** *p* < 0.0001 and ns = not significant).

Reactive astrogliosis is thought to prevent the regeneration of endogenous axons as well as the integration of transplanted NPCs in the spinal cord, hence being a key component of secondary injury changes after SCI, which we assessed by measuring the immuno-intensity of the astrocytic marker glial fibrillary acidic protein (GFAP) eight weeks after SCI (Figure 4A). NPC animals (group 1) had a slightly lower GFAP-immuno-intensity (18.03 ± 1.49 pi/mm^2^) in comparison to Vehicle animals (group 2; 22.04 ± 2.34 pi/mm^2^) without reaching a statistically significant difference (*p* = 0.2624; Figure 4B**)**. Nevertheless, compared to Sham animals (group 3; 13.02 ± 1.165 pi/mm^2^) astrogliosis was only significantly lower in Vehicle animals (*p* = 0.006) but not in NPC animals (*p* = 0.1365; Figure 4B).

The formation of a posttraumatic intramedullary cyst as another important pathophysiological process after SCI potentially reduces the amount of intact, preserved spinal cord tissue and might act as a physical barrier for axonal regeneration and sprouting. We, therefore, quantified the preserved spinal cord tissue in injured NPC (group 1) and Vehicle (group 2) animals as well as the intact spinal cord tissue in uninjured Sham animals (group 3) on consecutive spinal cord cross-sections stained for GFAP eight weeks after SCI. Animals in the NPC group had a significantly larger area of preserved spinal cord tissue (5.56 ± 0.32 mm^2^) compared to animals in the Vehicle group (4.5 ± 0.18 mm^2^; *p* = 0.0483; Figure 4C). As expected, the area of intact spinal cord tissue in uninjured Sham animals (7.14 ± 0.34 mm^2^) was significantly higher than the preserved spinal cord tissue in injured Vehicle (*p* < 0.0001) but also NPC animals (*p* = 0.0041; Figure 4C). In a more detailed analysis of the relatively preserved tissue at different distances to the lesion epicenter in injured animals, the beneficial effect of NPC-transplantation on tissue preservation could be predominantly observed in the caudal part with significantly more preserved tissue in the NPC group compared to the Vehicle group at 480 µm (62.8 ± 4.7% vs. 42.7 ± 3.6%; *p* = 0.0071) and 720 µm (74.5 ± 6.7% vs. 46.5 ± 7.3%; *p* = 0.0177; Figure 4D) caudal from the lesion.

These findings suggest that the transplantation of NPCs does not affect the extent of reactive astrogliosis around the lesion but the amount of preserved tissue more caudal to the lesion in the chronic phase after severe cervical SCI.

### 2.5. Tissue Scarring and Inflammation

**Figure 5:**
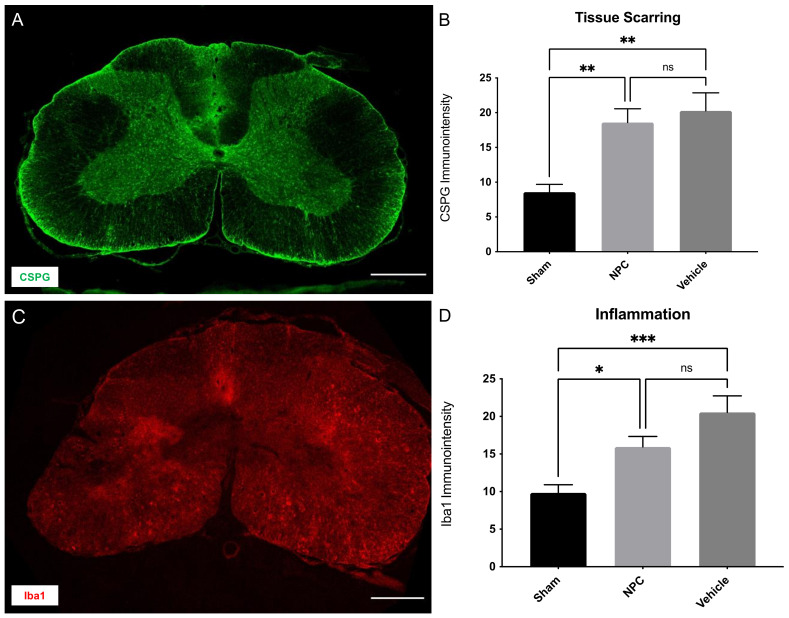
Tissue scarring and inflammation in the injured spinal cord eight weeks after severe cervical SCI. (**A**) Spinal cord cross-section of an NPC animal stained for CSPG, a marker for proteoglycans in the extracellular matrix at 10× magnification (scale bar = 500 µm). (**B**) The immuno-intensity of the CSPG-staining and thus the extent of tissue scarring showed no significant difference between NPC animals (group 1) and Vehicle animals (group 2; *n* = 8 animals per group; one-way ANOVA with Tukey-HSD-test; *p* = 0.8281). (**C**) Spinal cord cross-section of a Vehicle animal stained for Iba1, a marker for macrophages at 10× magnification (scale bar = 500 µm). (**D**) The immuno-intensity of the Iba1-staining and thus the number of Iba1^+^ macrophages was not significantly reduced in NPC animals compared to Vehicle animals (*n* = 8 animals per group; one-way ANOVA with Tukey-HSD-test; *p* = 0.1468) (* *p* < 0.05; ** *p* < 0.01; *** *p* < 0.001 and ns = not significant).

The accumulation of chondroitin sulfate proteoglycans (CSPGs) in the extracellular matrix of the injured spinal cord contributes to glial scar formation after SCI and acts as an additional physical barrier for axonal sprouting, hence preventing functional regeneration [23]. We, therefore, assessed the extent of tissue scarring by measuring the immuno-intensity of CSPG (Figure 5A) in the spinal cord eight weeks after the trauma. While CSPG-immuno-intensity was comparable between animals in the NPC and Vehicle group (18.59 ± 1.97 pi/mm^2^ in group 1 vs. 20.24 ± 2.607 pi/mm^2^ in group 2) and thus yielded no significant difference (*p* = 0.8281, Figure 5B), Sham animals (group 3; 8.58 ± 1.115 pi/mm^2^) showed significantly reduced tissue scarring in comparison to both, the NPC (*p* = 0.0067) and the Vehicle group (*p* = 0.0027; Figure 5B).

As SCI induces an inflammatory response, also persisting in chronic stages and potentially impeding neuroregeneration, we quantified the immuno-intensity of macrophages, identified by the marker ionized calcium-binding adaptor molecule 1 (Iba1), in the spinal cord eight weeks after trauma application (Figure 5C). Compared to uninjured Sham animals (9.84 ± 1.08 pi/mm^2^) the Iba1-immuno-intensity was significantly increased in both, the NPC (15.94 ± 1.4 pi/mm^2^; *p* = 0.0449) and the Vehicle animals (20.54 ± 2.195 pi/mm^2^; *p* = 0.0009; Figure 5D**)** in the chronic stage after SCI. However, while being slightly reduced with the NPC-treatment, the difference between the NPC and the Vehicle group remained unsignificant (*p* = 0.1468; Figure 5D).

We, therefore, conclude that NPC-transplantation does neither successfully reduce tissue scarring nor neuroinflammation in the cervical spinal cord eight weeks after severe SCI.

### 2.6. Functional Recovery

**Figure 6:**
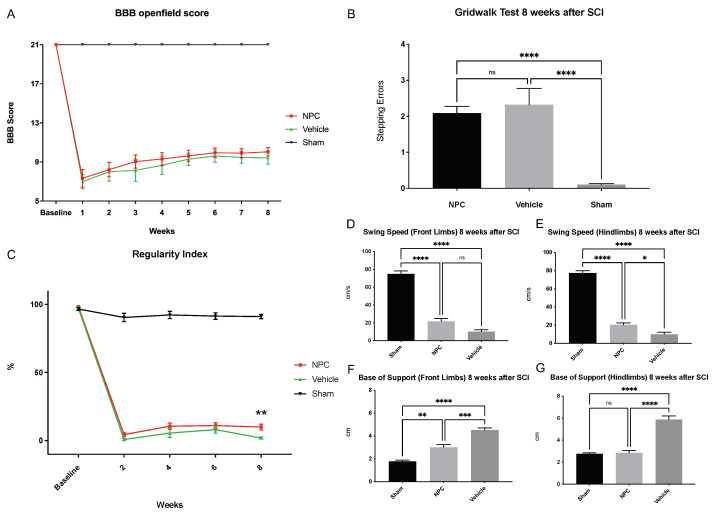
Functional recovery from baseline until eight weeks after severe cervical SCI. (**A**) While Sham animals (group 3; *n* = 8 animals) received 21 points on the BBB score at every timepoint, no significant difference in the BBB score could be observed between NPC animals (group 1) and Vehicle animals (group 2) throughout the experiment and recovery of injured animals generally remained low (*n* = 13 animals in the NPC group and *n* = 9 animals in the Vehicle group; two-way repeated measures ANOVA with Tukey test for multiple comparisons). (**B**) Similarly, assessment of fine motor control with the Gridwalk test revealed no significant difference between the NPC and the Vehicle animals at the end of the experiment (*n* = 13 NPC animals, *n* = 9 Vehicle animals and *n* = 8 Sham animals; one-way ANOVA with Tukey-HSD-test; *p* = 0.7984). (**C**) In contrast, the “Regularity Index” of the more objective CatWalk XT automated quantitative gait analysis as a measurement for coordination was significantly improved in NPC animals compared to Vehicle animals eight weeks after the injury (*n* = 13 NPC animals, *n* = 9 Vehicle animals and *n* = 8 Sham animals; two-way repeated measures ANOVA with Tukey test for multiple comparisons; *p* = 0.0051). Furthermore, while indifferent for the front limbs (**D**), the “Swing Speed” of the hindlimbs (**E**) in the CatWalk XT automated quantitative gait analysis was significantly improved in NPC animals compared to Vehicle animals eight weeks after SCI, suggesting less limping after NPC-transplantation (*n* = 13 NPC animals, *n* = 9. Vehicle animals and *n* = 8 Sham animals; Kruskal–Wallis test followed by Dunn’s multiple comparisons test; *p* = 0.0959 and *p* = 0.0158, respectively). Similarly, the “Base of Support” indicating trunk stability and weight-bearing when decreased was significantly lower in NPC animals compared to Vehicle animals at the end of the experiment in both, the front limbs (**F**) and the hindlimbs (**G**); *n* = 13 NPC animals, *n* = 9 Vehicle animals and *n* = 8 Sham; Kruskal–Wallis test followed by Dunn’s multiple comparisons test; *p* = 0.0006 and *p* < 0.0001, respectively) (* *p* < 0.05; ** *p* < 0.01; *** *p* < 0.001; **** *p* < 0.0001 and ns = not significant).

General locomotor recovery was assessed with the Bassso-Beatti-Bresnahan (BBB) score, which we performed at baseline and weekly after SCI. While the maximum BBB score (21 points) was achieved by all animals at baseline, it showed a drastic decline in all NPC and Vehicle animals one week after SCI (7.3 ± 0.9 vs. 7 ± 0.7 points). Although gradual improvement could be observed, the recovery of joint and hindlimb movements, stepping, front limb, and hindlimb coordination, paw placement as well as trunk and tail position as measured with the BBB score remained low throughout the experiment in both SCI groups. The highest score in NPC animals (group 1) was reached eight weeks post-SCI (10.04 ± 0.43 points vs. 9.42 ± 0.65 points in Vehicle animals; *p* = 0.7207), while the highest score in Vehicle animals (group 2) was already reached six weeks post SCI (9.61 ± 0.64 points vs. 9.94 ± 0.48 points in NPC animals; *p* = 0.9111). However, no injured animal showed frequent to consistent weight-supported plantar stepping with occasional front limb-hindlimb coordination (>11 points). Moreover, no statistically significant improvement could be observed with NPC-transplantation compared to untreated Vehicle animals at any timepoint (Figure 6A). In contrast, Sham animals (group 3) achieved the maximum BBB score at every timepoint, indicating a constantly and significantly better locomotor function compared to injured NPC or Vehicle animals (all *p* < 0.0001 from week 1 to week 8 after SCI).

Analysis of the Gridwalk test eight weeks after SCI revealed slightly improved fine motor control in NPC animals with 2.1 ± 0.19 stepping errors per run compared to Vehicle animals with 2.3 ± 0.45 stepping errors per run. However, this difference remained insignificant (*p* = 0.7984). As expected, animals in the Sham group had almost no stepping errors per run (0.1 ± 0.03) and thus displayed significantly better fine motor control compared to NPC (*p* < 0.0001) and Vehicle animals (*p* < 0.0001; Figure 6B).

As an objective complement to the BBB score, we additionally used the “Regularity Index” (RI) measurement in the CatWalk XT automated quantitative gait analysis system to assess general locomotor function and, more specifically, coordination. The RI is defined as the exclusive use of normal step sequence patterns during uninterrupted locomotion, and an animal with an RI of 100% is, therefore, regarded as fully coordinated. While all animals showed an RI of nearly 100% at baseline (NPC group: 98.3%; Vehicle group: 97.2% and Sham group: 96.6%; no significant differences), the RI of NPC and Vehicle animals had substantially decreased two weeks after SCI (4.43% vs. 0.81%; *p* = 0.3385) and remained low throughout the course of the experiment (week 4: 10.61% vs. 5.46%; *p* = 0.1234 and week 6: 11.05% vs. 8.1%; *p* = 0.4860). Nevertheless, eight weeks after SCI, the RI in the NPC group (9.97%) was significantly higher compared to the Vehicle group (1.89%; *p* = 0.0051; Figure 6C). Of note, the RI in uninjured Sham animals was subject to minor fluctuations as well, reaching from 90.4% (week 2) to 92.2% (week 4). At all postinjury timepoints, however, Sham animals displayed a significantly higher coordination compared to NPC or Vehicle animals (all *p* < 0.0001).

With the CatWalk XT, we were also able to assess further aspects of functional recovery separately for the front- and the hindlimbs, which is especially crucial in cervical SCI. The measurement “Swing Speed” (SS) thereby describes the speed of the respective limbs while having no contact with the floor and is therefore corresponding to a limping pattern when decreased. At the end of the experiment, SS of the front limbs, was faster in NPC animals compared to Vehicle animals (21.7 ± 3.16 cm/s vs.10.21 ± 2.18 cm/s) without reaching a significant difference (*p* = 0.0959; Figure 6D). Concerning the hindlimbs, however, a significant difference between the SS of the NPC (20.39 ± 2.02 cm/s) and the Vehicle animals (9.97 ± 2.32 cm/s; *p* = 0.0158) could be observed (Figure 6E). Sham animals displayed a significantly faster SS of both, the front (74.79 ± 3.365 cm/s) and the hindlimbs (77.28 ± 2.78 cm/s) compared to NPC (*p* < 0.0001) as well as Vehicle animals (*p* < 0.0001). Interestingly, the transplantation of NPCs had comparable effects on the limping pattern of the front and the hindlimbs (*p* = 0.6822). A significant impact of NPC-transplantation eight weeks after injury could also be observed on the “Base of Support” (BOS) measurement, which represents the distance between the front limbs or hindlimbs perpendicular to the direction of walking: Compared to the Vehicle animals, animals in the NPC group had a significantly decreased BOS of the front and also of the hindlimbs (3 ± 0.24 cm vs. 4.5 ± 0.2 cm and 2.8 ± 0.22 cm vs. 5.86 ± 0.33 cm; *p* = 0.0006 and *p* < 0.0001, respectively, Figure 6F,G), indicating overall improved trunk stability and weight-bearing. The effects of NPC-transplantation on the distribution of body weight were thereby significantly lower in the front limbs compared to the hindlimbs (*p* = 0.0001). In the Sham group, the BOS of the front limbs (1.78 ± 0.10 cm) was significantly smaller compared to the NPC (*p* = 0.0028) and the Vehicle group (*p* < 0.0001). However, while significantly smaller compared to the Vehicle group (*p* < 0.0001), the hindlimb BOS of Sham animals (2.77 ± 0.09 cm) showed no relevant difference to the one of NPC animals (*p* > 0.9999).

In summary, these findings in the highly sensitive CatWalk XT gait analysis indicate that the transplantation of NPCs can slightly affect coordination, limping of the hindlimbs as well as the front- and hindlimb mediated trunk stability after severe cervical SCI. However, potential beneficial effects of the NPC-treatment were not robust enough to have a significant impact on overall locomotor recovery and fine motor control as measured with the BBB score and the Gridwalk test.

## 3. Discussion

Transplantation of stem cells has been a significant treatment paradigm for SCI in the last decade, rapidly evolving from preclinical studies to clinical translation [19,24]. Recent disenchanting results have, however, led to a critical reappraisal of the SCI models used for the generation of preclinical data [25,26,27]. Thereby, the frequent application of sharp injuries such as hemisection or transection of the spinal cord, leading to substantially less gliotic scarring, inflammation, demyelination, and neuron loss compared to the contusion-compression injury typically seen in human SCI, has been identified as a major confounder [20,28]. Moreover, injuries in animal models have predominantly been performed in the thoracic spine, whereas the cervical spine is most commonly affected in human SCI patients [3]. Given that the cervical spine has a unique and complex cellular circuitry, usually resulting in a distinct loss of function after injury, extrapolation from results in thoracic SCI appears critical [20]. In our current study, we, therefore, have evaluated the neuroprotective and neuroregenerative effects of NPC-transplantation after a contusion-compression injury at the C6 level, following the urge of the scientific community to provide proof-of-efficacy for this treatment strategy in a clinically more relevant model [21,29]. 

As an indicator of the injury severity of the clip contusion-compression model used in our experiment, we observed high mortality of animals (45%), typically occurring early after the cervical SCI due to respiratory dysfunction. It must be noted that, mortality rates are rarely published for comparative studies in the literature. The few existing reports, however, typically depict similar or even higher mortality rates after cervical contusion-compression SCI in rats [30,31,32]. By describing the injury-associated mortality in our study, we provide further evidence for the seriousness of the clip contusion-compression model and hence underline its similarity to the cervical SCI seen in humans. This similarity is additionally supported by our findings on neurological function, which was greatly impaired in all injured animals directly after trauma and remained low until the end of the experiment, demonstrating the severe morbidity associated with cervical SCI even in the long run. As histological markers for the severity of the injury, we could observe considerable astrogliosis, tissue scarring, inflammation, and bilateral gray matter loss with the formation of cystic cavities eight weeks after SCI in our study. While these findings correspond well to previous reports on the clip contusion-compression model in the literature, we additionally provide evidence of reduced myelination, motor neurons, and ascending/descending tracts in the injured spinal cord, which are similar to long-term effects seen in human SCI patients [20,30,33,34].

Despite the verified severity of the SCI model used in our study, we were able to demonstrate long-term survival of NPCs that had been transplanted into the spinal cord ten days after the injury. Several factors hereby might have played a crucial role: Firstly, by transplanting NPCs in the subacute phase after SCI, we were aiming to avoid the early inflammatory response with, e.g., the release of inflammatory cytokines or infiltration of immune cells, which are considered to be neurotoxic for the cell graft [35,36]. Moreover, from a translational point of view, the subacute phase after SCI has additional benefits considering the practical and logistical aspects of cell-based treatment strategies [37,38]. On the other hand, transplantation of stem cells beyond the subacute phase has proven to be difficult and resulted in marginal or no functional benefits of the treatment in previous studies [10,14,39]. Secondly, because immune rejection by the host is a relevant limiting factor for the survival of the cell graft after transplantation, continuous immunosuppression was performed in all animals in our study, although donor and host were the same species [40]. Thirdly, we used osmotic micropumps to continuously administer the growth factors EGF, bFGF, and PDGF-AA, which have previously demonstrated beneficial effects on the survival and proliferation of NPCs, locally to the cell graft for seven days directly after transplantation [41,42,43].

Although the resulting long-term survival rate of NPCs in our study (1.4% of the initial cell graft/per animal) may seem low, it is in accordance with previous reports in the literature where even less severe trauma models have been used [44]. More importantly, improved neuroregeneration after SCI is likely not only dependent on the long-term survival of the transplanted stem cells, but also their early neuroprotective and modulatory effects via, e.g., the secretion or upregulation of trophic factors, which indirectly affect the injured spinal cord far beyond their death [45,46]. Nevertheless, integration of the cell graft into the host tissue and differentiation of the transplanted cells are thought to play a crucial role in functional recovery after SCI, especially when considering the tripotential capacity of NPCs [47]. In contrast to many transplantation paradigms in the literature, we could observe differentiation of transplanted NPCs eight weeks after severe cervical SCI predominantly into mature oligodendrocytes (46%) [48,49,50]. Differentiation into mature neurons, on the other hand, remained low (3%). This differentiation pattern of transplanted NPCs in our study might be related to the simultaneous intrathecal administration of the growth factor PDGF-AA, which has been associated with regulating the proliferative response of oligodendrocyte precursors and has been shown to foster the survival of newly formed oligodendrocytes [51,52]. Furthermore, there is growing evidence that the endogenous environment of the spinal cord might contain inhibitory factors for generating new neurons from NPCs, contributing to a low number of NPC-derived neurons present in the chronic injury stage in our study [14]. Of note, 35% of the surviving cell graft was still in an undifferentiated state eight weeks after the injury. Those NPCs could still be highly relevant for regeneration and repair via the secretion of, e.g., extracellular vesicles, which are thought to be involved in the guidance of axonal growth, the modulation of synaptic activity or the differentiation of other NPCs [53].

Accordingly, we could observe significantly more perilesional motor neurons in animals with NPC-transplantation, suggesting that the treatment has indirectly affected the preservation or regeneration of those endogenous cells, as has been reported by other authors [30,54]. Because the local circuitry in the cervical spinal cord, which is partly mediated by serotonergic projections from motor neurons, is crucial for functional recovery, this effect of transplanted NPCs might counterbalance their reduced neuronal differentiation [30]. Furthermore, although it has been postulated that improved neurological outcome seen after SCI in animal models is dependent upon neuronal differentiation of transplanted NPCs, which could form synapses with the injured axons or host interneurons themselves, oligodendroglial differentiation has been shown to independently improve functional recovery as well [37,55,56]. Potentially, NPC-derived oligodendrocytes replenish the loss of endogenous oligodendrocytes and consequently hinder the ongoing demyelination, which is said to be a major sequelae of SCI [22,57]. In our study, however, the transplantation of NPCs had neither significantly increased the degree of myelination nor the number of oligodendrocytes/actively myelin-producing oligodendrocytes in the injured spinal cord eight weeks after severe cervical SCI.

Correspondingly, descending tracts which typically suffer from demyelination after SCI were not found to be significantly preserved or regenerated across the posttraumatic lesion in the presence of NPCs after retrograde fiber labeling with FG. In combination with the observed low neuronal differentiation of NPCs, these findings could thus be associated with a corresponding lack of axon-oligodendrocyte interactions: Axons express neuronal factors such as neuregulin-1 which control oligodendrocyte function—their reduced presence after SCI despite the transplantation of NPCs might thus have caused the reduction in myelination, independent from the increased number of NPC-derived oligodendrocytes [58,59]. When assessing the total number of ascending and descending spinal tracts by anterograde transportation of Mn^2+^ across the lesion in MEMRI, we could, however, observe significantly improved overall spinal tract preservation or regeneration after NPC-transplantation. These findings suggest, that the integrity of spinal tracts which are deemed to be crucial for functional recovery after SCI is not only dependent on remyelination but rather on additional effects, possibly mediated by the transplanted NPCs [8].

Such additional effects might be the disruption of the gliotic scar, which is formed by reactive astrocytes, typically preventing axonal growth and neuronal plasticity, or the reduction of the posttraumatic cystic cavities, which are considered to be relevant obstacles for axonal regeneration and sprouting, especially in the chronic phase after SCI [33,60,61]. Corresponding to previous reports, the transplantation of NPCs had led to a significant increase of preserved grey and white perilesional matter eight weeks after cervical SCI in our study. However, we could not verify reported effects of the NPC graft on reactive astrogliosis in the injured tissue in the chronic stage after severe SCI [20]. Similarly, we did not observe a significantly reduced infiltration of macrophages into the damaged spinal cord which would have been of particular importance, because such inflammatory cells can continuously exacerbate the hostile postinjury microenvironment with their cellular products, including reactive oxygen species, proinflammatory cytokines, or matrix metalloproteinases and thus impede axonal regeneration [61,62].

Nevertheless, the observed neuropathological aspects of improved neuroregeneration were associated with improved functional recovery in NPC-treated animals as well: While the assessment of general locomotor function with the BBB score and fine motor control with the Gridwalk test only revealed non-significant positive effects of the NPC graft, the more objective and sensitive CatWalk XT analysis was able to identify significant improvements of gait coordination, strength and paw control in NPC-treated animals in the later stages after SCI. These differences could be explained by the ability of the CatWalk XT analysis to simultaneously record the contact of all four paws in slow-motion, allowing automatic and detailed assessment of front limb—hindlimb coordination and fine paw movement [63]. Therefore, a possible lack of sensitivity of the BBB score in assessing functional recovery after severe cervical SCI, which has already been postulated elsewhere, seems to be confirmed by our findings, questioning the necessity of this neurobehavioral test in comparable models in general [20,64]. Of note, although the BBB score allows for a comprehensive analysis of locomotion in general, it is unable to evaluate the front limbs directly, while behavioral tasks such as grip strength, rearing in the cylinder test or pellet-reaching/grasping while standing are not utilizable to evaluate front limb functional recovery in more severe injury models [20,65,66]. Neurobehavioral tests such as the CatWalk XT analysis are therefore of utmost importance in assessing severe cervical spinal contusion/compression injures. Although NPC-transplantation after SCI has been shown to enhance functional recovery in other studies, ours is one of the few that used the CatWalk XT analysis to provide more detailed evidence on the improved regeneration pattern of not only the hindlimbs but also the front limbs [20,30,33,34,67]. Indeed, we were able to detect a significantly higher “Base of Support” in the front limbs compared to the hindlimbs with the NPC-treatment, which implies increased overall trunk stability through hindlimb rather than front limb regeneration [68,69]. Improvement of “Swing Speed” and thus the limping pattern of the hindlimbs after NPC-transplantation was, however, comparable.

With our current study, we are thus able to demonstrate that long-term survival of transplanted NPCs and their subsequent direct, as well as indirect effects on secondary injury processes and neuropathological neuroregeneration do have an impact on functional recovery even after a severe and clinically relevant contusion/compression injury of the cervical spine. Given the low survival rate of the transplanted NPCs in our experiment as well as in comparable studies, their observed neuroprotective and neuroregenerative properties, even in such small quantities, are indeed remarkable [20,30,33,47,61]. While not assessed in our current experiment, it is noteworthy that transplanted NPCs have been associated with a reduction of neuropathic pain after SCI in animal models as well, possibly mediated by paracrine release of anti-inflammatory cytokines or the general attenuation of the cellular inflammatory response [70,71]. Future studies, therefore, should focus on synergistic treatment approaches, combining NPC-transplantation with, e.g., neuroprotective agents or interventions to increase survival and differentiation of the engrafted NPCs, which might considerably increase their neuroregenerative potential.

## 4. Materials and Methods

### 4.1. Isolation and Cultivation of NPCs

**Figure 7:**
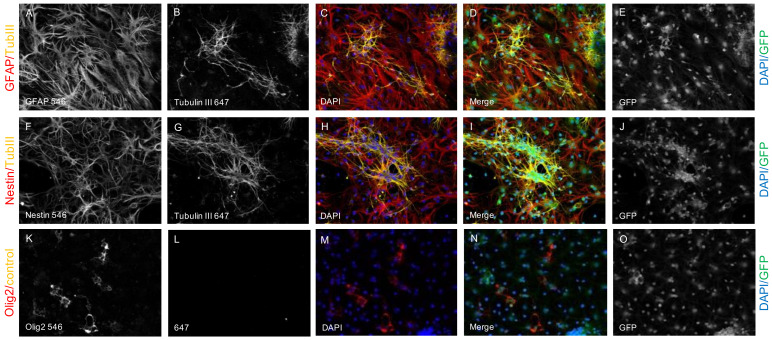
DAPI^+^ (blue) and GFP^+^ (green) cells are expressing Nestin ((**F**), red) and are thus identified as viable NPCs ((**I**), green/blue/red). The tripotential differentiation capacity of the NPCs is assessed by costaining of GFP ((**E**,**J**,**O**,**D**,**I**,**N**), green) and DAPI ((**C**,**H**,**M**,**D**,**I**,**N**), blue) with TubIII ((**B**,**G**,**C**,**H**,**D**,**I**), yellow), GFAP ((**A**,**C**,**D**), red) and Olig2 ((**K**,**M**,**N**), red) indicating viable NPC-derived neurons ((**D**,**I**), green/blue/yellow), viable NPC-derived astrocytes ((**D**,**I**), green/blue/red) and viable NPC-derived oligodendrocytes ((**N**), green/blue/red). respectively. Only the secondary antibody (Alexa-647) has been used as a negative control (**L**).

NPCs were isolated from the subventricular zone of 2-week-old embryos (E14) of green fluorescent protein (GFP) expressing transgenic Wistar rats (Rat Resource & Research Center, University of Missouri, Columbia, MO, USA; strain F344-Tg(UBC-EGFP)F455Rrrrc) as previously described [61]. In short, the cortical hemispheres of the embryos were carefully dissected, freed from meninges, and washed in 2 mL cold PBS (without CaCl_2_ and MgCl_2_; Thermo Fisher, Waltham, MA, USA). After removal of the buffer, 1.5 mL 0.05% trypsin/ethylenediaminetetraacetic acid with 0.2% deoxyribonuclease I (Thermo Fisher, USA) was added to the tissue pieces and the suspension was incubated at 37 °C for 5 min before inhibition of the enzymatic activity with 10% fetal bovine serum (Thermo Fisher, USA). The tissue was then mechanically dissociated into a cell suspension with a fire-polished pipette and finally centrifuged at 1200× *g* rpm for 6 min. For cultivation, NPCs were plated on poly-l-ornithine laminin-coated tissue culture plates (Sigma-Aldrich, St. Louis, MO, USA) at a density of 1.5 × 10^4^ cells/cm^2^ in 1.5 mL growth medium containing Dulbecco’s Modified Eagle’s Medium (DMEM)/_F12_ with sodium bicarbonate and L-glutamine (Thermo Fisher, USA), 1% penicillin/streptomycin, 1 × N2 supplement (both Gibco, Waltham, MA, USA), 20 ng/mL bFGF, and 20 ng/mL EGF (both Sigma-Aldrich, St. Louis, MO, USA). NPCs were incubated in a humidified incubator at 37 °C with 5% CO_2_ and split from 1:3 to 1:6 when a confluence of 80–90% was reached. The viability of the NPCs, as well as their stem cell characteristics and tripotential differentiation capacity into all three neural cell types were successfully assessed before further use (Figure 7). To this end, the cells on culture plates were fixed with paraformaldehyde (4% in 0.1 M PBS at pH 7.4) for 20 min and were then subjected to immunofluorescence staining.

### 4.2. Animals, Experimental Groups and Study Design

**Figure 8:**
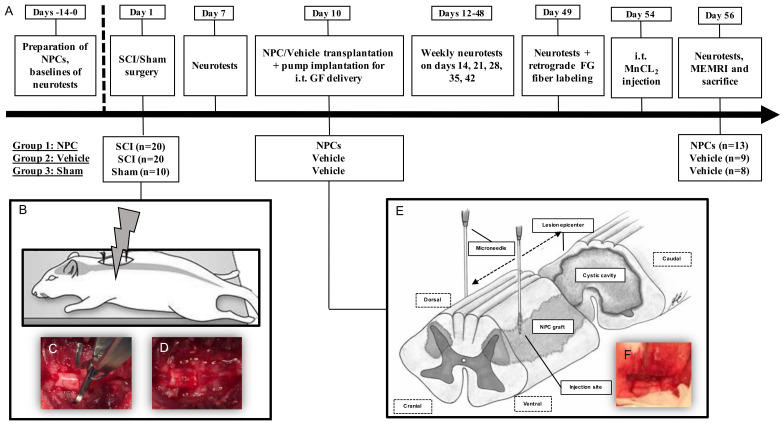
(**A**) Timeline of the study interventions (e.g., pump implantation for i.t. growth factor (GF) delivery at day 10 or manganese-enhanced magnetic resonance imaging (MEMRI) at day 56), also indicating the group design and the group sizes at the beginning and at the end of the experiment. (**B**) The contusion-compression SCI is performed at day 1 with a modified aneurysm clip (**C**), leaving a visible injury on the exposed cervical spinal cord at the C6 level (**D**). (**E**) Modified after (47)—NPCs are transplanted into the spinal cord at four distinct sites via stereotactic injection (**F**) at day 10.

A total of 50 female Wistar rats (250 g; Charles River Laboratories, Sulzfeld, Germany), randomly assigned to three treatment groups were used for this experiment: Group 1 (NPC; *n* = 20), group 2 (Vehicle; *n* = 20) and group 3 (Sham; *n* = 10). Assignment for groups 1 and 2 took place directly after SCI. NPCs were transplanted 10 days after the injury and intrathecal administration of growth factors was simultaneously initiated. For immunosuppression, minocycline (50 mg/kg s.c.; Ratiopharm, Ulm, Germany) was administered daily from two days prior to 10 days after the transplantation and daily injections of ciclosporin (10 mg/kg s.c.; Sigma-Aldrich, St. Louis, MO, USA) were initiated on the day of transplantation and continued until the end of the experiment in all groups. Neurotests for the assessment of functional outcome were performed weekly, and the experiment was terminated with the perfusion of all animals eight weeks after SCI (Figure 8A). All surgeries and outcome assessments were blinded, and all experimental protocols were approved by the Animal Care Committee of the federal government of Baden Württemberg, Germany (ethic approval code G-211/15, 21 September 2015). Morbidity leading to sacrifice of selected animals prior to study termination as well as early mortality due to the severity of the cervical injury were high and resulted in a reduced number of animals available for analysis in groups 1 (NPC; *n* = 13) and 2 (Vehicle; *n* = 9). In group 3 (Sham; *n* = 8), two animals had to be excluded due to unintentional injury to the spinal cord during laminectomy.

### 4.3. Spinal Cord Injury and NPC-Transplantation

For all surgical procedures, animals were anesthetized with isoflurane (1.5–2.5%) and a 1:1 mixture of O_2_ and N_2_O. We used a 28 g modified aneurysm clip (Fehlings Laboratory, Toronto, ON, Canada) to induce a cervical contusion-compression SCI at the C6 level in NPC and Vehicle animals (groups 1 and 2), as previously described [20,72]. In short, a C6 and C7 laminectomy was performed, the clip was applied around the cord, snap shut, and sustained for 60 s by the same surgeon in all animals (Figure 8B–D). Animals in the Sham group (group 3) received a laminectomy of C6 and C7 only. Postoperatively, all animals were subjected to extensive care and received buprenorphine (0.05 mg/kg s.c.; Bayer, Leverkusen, Germany) as well as meloxicam (2 mg/kg s.c.; Boehringer-Ingelheim, Ingelheim, Germany) for 3–5 days. Fluids and nutritional support were administered, antibiotic prophylaxis (moxifloxacin, 4 mg/kg p.o.; Alcon, Fort Worth, TX, USA) was given for seven days, and bladders were manually expressed three times per day until reflexive bladder function had recovered. Animals were housed in a 12 h light-dark cycle at 26 °C with food and water ad libitum.

For transplantation of NPCs 10 days after SCI, the dura at the C6 level was microsurgically re-exposed. Then, 4 × 10^5^ NPCs at passage three (P3) diluted in 8 µL (i.e., 2 µL per site) growth medium were injected into the spinal cord at four sites, bilaterally 2 mm rostral and caudal to the epicenter of the lesion at a depth of 1.5 mm, using a stereotactic microinjector with a Hamilton syringe (Hamilton Company, Bonaduz, Switzerland) and 35 G microneedle (World Precision Instruments, Sarasota, FL, USA) at a rate of 5 nL/s (Figure 8E) [33]. Animals in group 2 (Vehicle) received the same amount of growth medium without NPCs and in sham animals (group 3), the dura was only re-exposed, and no stereotactic injection was given. During the same surgery, a skip-laminectomy of T1 was performed, and a rat intrathecal microcatheter (Alzet, Cupertino, CA, USA), connected to a subcutaneous osmotic micropump (model 1007D; Alzet, Cupertino, CA, USA) was subdurally placed with its open tip over the epicenter of the lesion as described previously [73]. This pump was used for continuous intrathecal administration of growth factors (PDGF-AA, 1 µg/100 µL; EGF, 3 µg/100 µL; bFGF, 3 µg/100 mL; all Sigma-Aldrich, St. Louis, MO, USA), diluted in 0.1% rat serum albumin (Sigma-Aldrich, St. Louis, MO, USA) in all injured animals (group 1 and 2) at 0.5 µL/h for 7 days [61]. Sham animals (group 3) received a T1 skip-laminectomy only.

### 4.4. Behavioral Assessment

The Basso–Beattie–Bresnahan locomotor rating scale (BBB) was performed weekly in all animals to assess functional recovery over the course of the experiment. To this end, rats were placed into an open field for 4 min, and hindlimb locomotor function, joint movement, coordination as well as weight bearing were evaluated by three independent observers using a rating scale from 0 to 21 points [74]. In addition, all animals underwent a CatWalk XT automated quantitative gait analysis (CatWalk XT, Noldus Ltd., Wageningen, Netherlands) every two weeks after SCI. Thereby, a minimum of three nonstop runs over the CatWalk XT glass walkway was recorded at each timepoint while by a camera placed under the glass floor. Briefly, light from a fluorescent tube is sent through the glass floor and is reflected downwards as soon as the animal’s paw is in contact with the surface, resulting in a sharp image of a paw print [75]. Prints were automatically classified with the CatWalk XT software (atWalk XT^®^ version 8.1; Noldus Ltd., Wageningen, Netherlands) and additional manual examination of mislabeled or irrelevant prints was performed. Specific, automatically calculated parameters for front- and hindlimb function as well as movement pattern (print area, swing speed, base of support, regularity index) were obtained. Animals that did not show constant weight-bearing stepping were also included in this analysis. Furthermore, on the last day of the experiment eight weeks after SCI, fine motor coordination was specifically assessed using the Gridwalk test as an endpoint measurement. In short, step errors of animals crossing a pathway of irregularly placed metal grids were counted over four runs by three independent observers, and errors were averaged [76]. Baselines for the BBB, the CatWalk XT, and the Gridwalk test were acquired prior to SCI for all animals.

### 4.5. Retrograde Fiber Labeling and Manganese-Enhanced MR-Imaging

Retrograde fiber labeling with FluoroGold (FG; Fluorochrome, Denver, CO, USA) was performed seven days prior to the end of the experiment in five randomly selected animals per group. To this end, a T2 laminectomy was completed, the dura was exposed, and two injections of 0.5 µL FG (4%, dissolved in PBS) were given stereotactically-guided, 0.5 mm bilateral to the posteromedian vein at a depth of 1.2 mm using the same set-up as described above. FG is migrating through the axons across the lesion site and can be visualized in the cell bodies of neurons located in the brainstem seven days after injection, thus serving as a surrogate marker for the amount of intact descending tracts in the injured spinal cord [77].

In addition, the same five animals per group underwent in vivo manganese-enhanced magnetic resonance imaging (MEMRI) on the last day of the experiment immediately before perfusion. Mn^2+^ is a paramagnetic ion which resembles Ca^2+^ and leads to substantial contrast enhancement in T1-weighted MRI [78]. Because of its active uptake into intact neurons via voltage-gated Ca^2+^ channels [79], further anterograde transport along the axon in a microtubule-based fashion and crossing of active synapses [80,81], it has been successfully used as a contrast agent for intact neural tissue in the injured spinal cord after rodent SCI [82]. Intrathecal application of Mn^2+^ was performed 48 h prior to scanning. To this end, animals were anesthetized, placed in a stereotactic frame with a 90° decline of the head, and 80 µL of 0.8 mM MnCl_2_-solution were manually injected into the cisterna magna via the membrana atlanto-occipitalis using a 27 g needle [83]. Two days later, a clinical 1.5-T scanner (Siemens Symphony, Erlangen, Germany) with a custom-made animal volume resonator was used to employ a T1-weighted 3-dimensional FLASH imaging pulse sequence (TR/TE 14.0/5.22 ms, voxel size in-plane 0.15 mm × 0.15 mm, 16 averages, flip-angle 30°, 28 partitions, partition thickness 0.5 mm, the field of vision 80 mm, matrix size 512 × 512, scan time 30 min) and images were taken in the sagittal plane for positioning and in the axial plane for detailed measurements [82]. Images were evaluated using the MTIK software, and the spinal cord was divided into axial slices from 4 mm rostral to 4 mm caudal to the lesion epicenter in injured or the C6 vertebra in sham animals. Signal-to-noise ratio (SNR) was then measured in every axial slice by placing a region of interest (ROI) outside the animal contours for noise measurement and a second ROI around the spinal cord. SNR is either given as the mean overall value for the cervical and thoracic spinal cord or separately as the mean rostral and mean caudal value per group [84].

### 4.6. Animal Perfusion and Tissue Processing

At the end of the experiment, eight weeks after SCI, animals were deeply anesthetized with isoflurane (4%) and transcardially perfused with 50 mL 0.1 M cold phosphate-buffered saline (PBS) followed by 150 mL paraformaldehyde (4% in 0.1 M PBS at pH 7.4). Brains and spinal cords were removed, post-fixed in 4% paraformaldehyde for 24 h, and cryoprotected in 30% sucrose for 48 h. Segments of the spinal cord with a length of 2 cm, centered around the lesion epicenter were dissected and embedded in tissue embedding medium (Sakura Finetek Europa B.V., Alphen aan den Rijn, Netherlands) on dry ice. A cryostat (Leica Biosystems, Nussloch, Germany) was then used to cut consecutive cross-sections of the spinal cord segments (every 240 µm) and the brains (continuously) with a thickness of 30 µm and 25 µm, respectively. All tissue sections were dried and stored at −80 °C for further processing.

### 4.7. Immunofluorescence Staining

For immunofluorescence staining, NPCs on culture plates or spinal cord sections were first blocked with a blocking solution containing 5% non-fat milk powder, 1% bovine serum albumin, and 0.3% Triton-X100 in 0.1 M PBS (all Sigma-Aldrich, St. Louis, MO, USA) for 1 h at room temperature and then incubated with the following primary antibodies, diluted in the same blocking solution at 4 °C overnight: anti-Nestin (1:200; Merck-Millipore, Darmstadt, Germany) for undifferentiated NPCs, anti-TubIII (1:250; Merck-Millipore, Germany) or anti-NeuN (1:500; Merck-Millipore, Germany) for neurons, anti-ChaT (1:100; Merck-Millipore, Germany) for motor neurons, anti-APC (1:200; Merck-Millipore, Germany) or anti-Olig2 (1:200; Merck-Millipore, Germany) for mature oligodendrocytes, anti-MBP (1:100; Santa Cruz Biotechnology, Dallas, TX, USA) for myelin basic protein (MBP)/myelination, anti-GFAP (1:1000; Merck-Millipore, Germany) for astrocytes/astrogliosis, anti-CSPG (1:400; Merck-Millipore, Germany) for chondroitin sulfate proteoglycans (CSPGs)/tissue scarring and anti-Iba1 (1:1000; Thermo Fisher Scientific, USA) for macrophages/inflammation. Isotype controls with non-specific immunoglobulin at the same concentration were performed to ensure the specificity of the antibody. Alexa Fluor 546 goat anti-rabbit (1:500; Thermo Fisher Scientific, USA), Alexa Fluor 568 goat anti-mouse (1:500; Thermo Fisher Scientific, USA), Alexa Fluor 647 goat anti-rabbit (1:500; Thermo Fisher Scientific, USA), and/or Alexa Fluor 405 donkey anti-goat (1:400; Abcam, Boston, MA, USA) diluted in blocking solution without Triton-X100 were used as secondary antibodies and applied for 1 h at room temperature. If applicable, DAPI (1:10,000; Sigma-Aldrich, St. Louis, MO, USA) was added for 30 min before washing the NPCs on culture plates with PBS three times or covering the sections with mounting medium. For brain cross-sections with FG labeling, no additional staining was necessary before mounting.

### 4.8. Imaging Analysis

Images of the NPCs on culture plates or the tissue sections were obtained using a confocal laser scanning microscope (LSM 700, Carl-Zeiss, Jena, Germany) at 10× magnification in the 8-bit-format with the tile scan function (speed of six, gain of 800). Five different wavelength-channels (DAPI-405nm, GFP-488 nm, Alexa Fluor 546 nm, Alexa Fluor 568 nm, Alexa Fluor 647 nm) with light transmission ranging from 2.8% to 100% were used.

The qualitative in vitro assessment of NPC viability (GFP^+^/DAPI^+^/Nestin^+^ cells) and NPC differentiation capacity (GFP^+^/DAPI^+^/TubIII^+^ NPC-derived neurons, GFP^+^/DAPI^+^/GFAP^+^ NPC-derived astrocytes and GFP^+^/DAPI^+^/Olig2^+^ NPC-derived oligodendrocytes) was performed in triplicates and representative images were additionally taken at 40× magnification (speed of four).

All quantitative in vivo imaging analyses were performed in eight randomly selected animals per group. For the quantitative assessment of NPC survival (GFP^+^ cells) and differentiation (GFP^+^/NeuN^+^ cells or GFP^+^/APC^+^ cells), spared motor neurons (ChaT^+^ cells) and myelin producing oligodendrocytes (MPB^+^/Olig2^+^ cells), semi-automatic cell counting was performed on cross-sections −4800 µm to +4800 µm from the lesion epicenter by three independent investigators blinded to treatment groups. To this end, we used an algorithm for ImageJ2 (National Institute of Health, Bethesda, MD, USA) as previously described [61]. Briefly, the images were split into single channels, a Gaussian-filter (Sigma: 10.00) was applied to reduce background noise, and the IsoData-thresholding algorithm was used to transform a selected region of interest (ROI) into a binary image. ROIs consisted of the entire spinal cord without the cyst and the autofluorescence border. Positive-labeled cells with signals above specific thresholds were then counted in the selected ROI with the “Analyze Particles” function. Next, binary images were recombined using the “Image Calculator” function, and the co-stained cells within the same ROI were counted. To avoid the inclusion of artifacts, only structures with an area of 50–2000 µm^2^ were considered. The total number of positively labeled cells was estimated by multiplying the total cell count of all consecutive cross-sections with the inter-section distance (240 µm) divided by the section thickness (30 µm). Results are given as the total number of cells (NPC survival and differentiation, spared motor neurons, oligodendrocytes and myelin producing oligodendrocytes).

For the assessment of axonal regeneration after retrograde fiber-labeling, the same method was used, but ROIs were placed around the whole brainstem on consecutive brain cross-sections, and FG^+^ cells were counted separately. The total number of FG^+^ cells was then calculated as the sum of the counting-result on all cross-sections within the whole brainstem.

For the quantitative assessment of myelination (MBP), astrogliosis (GFAP), tissue scarring (CSPG), and inflammation (Iba1), the immuno-intensity of the respective antibody was quantified on five distinct tissue sections at different distances from the lesion epicenter (0 µm, +/− 1200 μm and +/− 2400 μm). To this end, images were split into single channels with the ImageJ2 software, ROIs were drawn around the entire spinal cord with the exclusion of the cyst as well as the autofluorescence border, and the “Measure” function was used to output the respective immuno-intensity (pixel intensity(pi)/mm^2^).

For the assessment of preserved tissue, 11 spinal-cord cross-sections stained for GFAP with distinct distances from the lesion epicenter (0 µm, +/− 240 µm, +/− 480 µm, +/− 720 µm, +/− 960 µm, and +/− 1200 μm) were evaluated in NPC and Vehicle animals with the ImageJ2 software. Hereby, a ROI was drawn around the entire spinal cord and another ROI around the cystic cavity on each cross-section and the “Measure” function was used to output the area of the preserved spinal cord tissue without the cyst. Of note, this measurement does not allow for detailed characterization of the preserved spinal cord tissue’s cells. Results are given as the mean absolute preserved tissue area (in mm^2^) and as the mean relative preserved tissue area (divided by the entire spinal cord area) for the NPC and Vehicle group.

For qualitative assessment of tissue- or cell-morphology, additional images were taken at 40× magnification (speed of four).

### 4.9. Statistical Analysis

All results are given as mean ± standard error of the mean (SEM). For the statistical comparison of BBB and CatWalk Walk XT RI results between groups and time points, a two-way repeated measure ANOVA, followed by a Tukey test for multiple comparisons was used. Means between multiple groups in the remaining neurobehavioral tests or neuroanatomical studies were analyzed using one-way repeated measures ANOVAs followed by post-hoc Tukey-HSD-tests (parametric) or Kruskal–Wallis tests followed by Dunn’s multiple comparisons tests (non-parametric). For the comparison of means between two groups, unpaired two-sample *t*-tests were used. Normality assumption was confirmed prior to all parametric analyses using Shapiro–Wilk normality tests, and a *p*-value of *p* < 0.05 was considered significant. All statistical analyses were performed using the software Prism (version 7.0, GraphPad Software, San Diego, CA, USA).

## Data Availability

The data presented in this study are available on reasonable request from the corresponding author.

## Abbreviations

ANOVA	Analysis of variance
APC	Adenomatous polyposis coli
BBB	Basso–Beattie–Bresnahan locomotor rating scale
bFGF	Basic fibroblast growth factor
BOS	Base of support
ChaT	Choline acetyltransferase
CSPG	Chondroitin sulfate proteoglycan
DMEM	Dulbecco’s Modified Eagle’s Medium
EGF	Epidermal growth factor
FG	FluoroGold
GF	Growth factor
GFAP	Glial fibrillary acidic protein
Iba1	Ionized calcium binding adaptor molecule 1
MBP	Myelin basic protein
MEMRI	Manganese-enhanced magnetic resonance imaging
MRI	Magnetic resonance imaging
NeuN	Neuronal nuclei
NPC	Neuronal precursor cell
Olig2	Oligodendrocyte transcription factor
PBS	Phosphate buffered saline
PDGF-AA	Platelet-derived growth factor AA
RI	Regularity index
ROI	Region of interest
SCI	Spinal cord injury
SEM	Standard error of the mean
SNR	Signal-to-noise ratio
SS	Swing speed
TubIII	*βIII-*Tubulin

## References

[1] Furlan J.C., Sakakibara B.M., Miller W.C., Krassioukov A.V. (2013). Global incidence and prevalence of traumatic spinal cord injury. Can. J. Neurol. Sci..

[2] Sekhon L.H., Fehlings M.G. (2001). Epidemiology, demographics, and pathophysiology of acute spinal cord injury. Spine.

[3] Furlan J.C., Fehlings M.G. (2009). The impact of age on mortality, impairment, and disability among adults with acute traumatic spinal cord injury. J. Neurotrauma.

[4] Anderson K.D. (2004). Targeting recovery: Priorities of the spinal cord-injured population. J. Neurotrauma.

[5] Anderson K.D., Sharp K.G., Steward O. (2009). Bilateral cervical contusion spinal cord injury in rats. Exp. Neurol..

[6] Tator C.H. (1995). Update on the pathophysiology and pathology of acute spinal cord injury. Brain Pathol..

[7] Wu J., Pajoohesh-Ganji A., Stoica B.A., Dinizo M., Guanciale K., Faden A.I. (2012). Delayed expression of cell cycle proteins contributes to astroglial scar formation and chronic inflammation after rat spinal cord contusion. J. Neuroinflamm..

[8] Lu P., Wang Y., Graham L., McHale K., Gao M., Wu D., Brock J., Blesch A., Rosenzweig E.S., Havton L.A. (2012). Long-distance growth and connectivity of neural stem cells after severe spinal cord injury. Cell.

[9] Meletis K., Barnabé-Heider F., Carlén M., Evergren E., Tomilin N., Shupliakov O., Frisén J. (2008). Spinal cord injury reveals multilineage differentiation of ependymal cells. PLoS Biol..

[10] Assinck P., Duncan G.J., Hilton B.J., Plemel J.R., Tetzlaff W. (2017). Cell transplantation therapy for spinal cord injury. Nat. Neurosci..

[11] Tetzlaff W., Okon E.B., Karimi-abdolrezaee S., Hill C.E., Sparling J.S., Plemel J.R., Plunet W.T., Tsai E.C., Baptiste D., Smithson L.J. (2011). A systematic review of cellular transplantation therapies for spinal cord injury. J. Neurotrauma.

[12] Quertainmont R., Cantinieaux D., Botman O., Sid S., Schoenen J., Franzen R. (2012). Mesenchymal stem cell graft improves recovery after spinal cord injury in adult rats through neurotrophic and pro-angiogenic actions. PLoS ONE.

[13] Nakajima H., Uchida K., Guerrero A.R., Watanabe S., Sugita D., Takeura N., Yoshida A., Long G., Wright K.T., Johnson W.E.B. (2012). Transplantation of mesenchymal stem cells promotes an alternative pathway of macrophage activation and functional recovery after spinal cord injury. J. Neurotrauma.

[14] Karimi-Abdolrezaee S., Eftekharpour E., Wang J., Morshead C.M., Fehlings M.G. (2006). Delayed transplantation of adult neural precursor cells promotes remyelination and functional neurological recovery after spinal cord injury. J. Neurosci..

[15] Hawryluk G.W.J., Spano S., Chew D., Wang S., Erwin M., Chamankhah M., Forgione N., Fehlings M.G. (2014). An examination of the mechanisms by which neural precursors augment recovery following spinal cord injury: A key role for remyelination. Cell Transpl..

[16] Sankavaram S.R., Hakim R., Covacu R., Frostell A., Neumann S., Svensson M., Brundin L. (2019). Adult Neural Progenitor Cells Transplanted into Spinal Cord Injury Differentiate into Oligodendrocytes, Enhance Myelination, and Contribute to Recovery. Stem Cell Rep..

[17] Yousefifard M., Rahimi-Movaghar V., Nasirinezhad F., Baikpour M., Safari S., Saadat S., Moghadas Jafari A., Asady H., Razavi Tousi S.M.T.T., Hosseini M. (2016). Neural stem/progenitor cell transplantation for spinal cord injury treatment; A systematic review and meta-analysis. Neuroscience.

[18] Curtis E., Martin J.R., Gabel B., Sidhu N., Rzesiewicz T.K., Mandeville R., Van Gorp S., Leerink M., Tadokoro T., Marsala S. (2018). A First-in-Human, Phase I Study of Neural Stem Cell Transplantation for Chronic Spinal Cord Injury. Cell Stem Cell.

[19] Silvestro S., Bramanti P., Trubiani O., Mazzon E. (2020). Stem cells therapy for spinal cord injury: An overview of clinical trials. Int. J. Mol. Sci..

[20] Wilcox J.T., Satkunendrarajah K., Zuccato J.A., Nassiri F., Fehlings M.G. (2014). Neural precursor cell transplantation enhances functional recovery and reduces astrogliosis in bilateral compressive/contusive cervical spinal cord injury. Stem Cells Transl. Med..

[21] Kwon B.K., Soril L.J.J.J., Bacon M., Beattie M.S., Blesch A., Bresnahan J.C., Bunge M.B., Dunlop S.A., Fehlings M.G., Ferguson A.R. (2013). Demonstrating efficacy in preclinical studies of cellular therapies for spinal cord injury—How much is enough?. Exp. Neurol..

[22] Kim B.G., Hwang D.H., Lee S.I., Kim E.J., Kim S.U. (2007). Stem cell-based cell therapy for spinal cord injury. Cell Transpl..

[23] Siebert J.R., Osterhout D.J. (2011). The inhibitory effects of chondroitin sulfate proteoglycans on oligodendrocytes. J. Neurochem..

[24] Barnabé-Heider F., Frisén J. (2008). Stem cells for spinal cord repair. Cell Stem Cell.

[25] Oh S.K., Choi K.H., Yoo J.Y., Kim D.Y., Kim S.J., Jeon S.R. (2016). A Phase III Clinical Trial Showing Limited Efficacy of Autologous Mesenchymal Stem Cell Therapy for Spinal Cord Injury. Neurosurgery.

[26] Levi A.D., Anderson K.D., Okonkwo D.O., Park P., Bryce T., Kurpad S.N., Aarabi B., Hsieh J., Gant K. (2019). Clinical Outcomes from a Multi-Center Study of Human Neural Stem Cell Transplantation in Chronic Cervical Spinal Cord Injury. J. Neurotrauma.

[27] Lammertse D.P., Jones L.A.T., Charlifue S.B., Kirshblum S.C., Apple D.F., Ragnarsson K.T., Falci S.P. (2012). Autologous incubated macrophage therapy in acute, complete spinal cord injury: Results of the phase 2 randomized controlled multicenter trial. Spinal Cord.

[28] MM S., MK T., HS K. (2007). The Extent of Myelin Pathology Differs Following Contusion and Transection Spinal Cord Injury. J. Neurotrauma.

[29] Kwon B.K., Hillyer J., Tetzlaff W. (2010). Translational research in spinal cord injury: A survey of opinion from the SCI community. J. Neurotrauma.

[30] Iwasaki M., Wilcox J.T., Nishimura Y., Zweckberger K., Suzuki H., Wang J., Liu Y., Karadimas S.K., Fehlings M.G. (2014). Synergistic effects of self-assembling peptide and neural stem/progenitor cells to promote tissue repair and forelimb functional recovery in cervical spinal cord injury. Biomaterials.

[31] Moonen G., Satkunendrarajah K., Wilcox J.T., Badner A., Mothe A., Foltz W., Fehlings M.G., Tator C.H. (2016). A New Acute Impact-Compression Lumbar Spinal Cord Injury Model in the Rodent. J. Neurotrauma.

[32] Simard J.M., Tsymbalyuk O., Keledjian K., Ivanov A., Ivanova S., Gerzanich V. (2012). Comparative effects of glibenclamide and riluzole in a rat model of severe cervical spinal cord injury. Exp. Neurol..

[33] Zweckberger K., Ahuja C.S., Liu Y., Wang J., Fehlings M.G. (2016). Self-assembling peptides optimize the post-traumatic milieu and synergistically enhance the effects of neural stem cell therapy after cervical spinal cord injury. Acta Biomater..

[34] Karimi-Abdolrezaee S., Eftekharpour E., Wang J., Schut D., Fehlings M.G. (2010). Synergistic effects of transplanted adult neural stem/progenitor cells, chondroitinase, and growth factors promote functional repair and plasticity of the chronically injured spinal cord. J. Neurosci..

[35] Yasuda A., Tsuji O., Shibata S., Nori S., Takano M., Kobayashi Y., Takahashi Y., Fujiyoshi K., Hara C.M., Miyawaki A. (2011). Significance of remyelination by neural stem/progenitor cells transplanted into the injured spinal cord. Stem Cells.

[36] Anwar M.A., Al Shehabi T.S., Eid A.H. (2016). Inflammogenesis of secondary spinal cord injury. Front. Cell. Neurosci..

[37] Parr A.M., Kulbatski I., Zahir T., Wang X., Yue C., Keating A., Tator C.H. (2008). Transplanted adult spinal cord-derived neural stem/progenitor cells promote early functional recovery after rat spinal cord injury. Neuroscience.

[38] Lepore A.C., Han S.S.W., Tyler-Polsz C.J., Cai J., Rao M.S., Fischer I. (2004). Differential fate of multipotent and lineage-restricted neural precursors following transplantation into the adult CNS. Neuron Glia Biol..

[39] Keirstead H.S., Nistor G., Bernal G., Totoiu M., Cloutier F., Sharp K., Steward O. (2005). Human embryonic stem cell-derived oligodendrocyte progenitor cell transplants remyelinate and restore locomotion after spinal cord injury. J. Neurosci..

[40] Anderson A.J., Haus D.L., Hooshmand M.J., Perez H., Sontag C.J., Cummings B.J., Gross B., Cell S., Road H.S., Irvine U.C. (2012). Achieving stable human stem cell engraftment and survival in the CNS. Regen. Med..

[41] Lachapelle F., Avellana-Adalid V., Nait-Oumesmar B., Baron-Van Evercooren A. (2002). Fibroblast growth factor-2 (FGF-2) and platelet-derived growth factor AB (PDGF AB) promote adult SVZ-derived oligodendrogenesis in vivo. Mol. Cell. Neurosci..

[42] Shitaka Y., Saito H. (1994). The effect of basic fibroblast growth factor (bFGF) and nerve growth factor (NGF) on the survival of septal neurons transplanted into the third ventricle in rats. Jpn. J. Pharmacol..

[43] Vescovi A.L., Reynolds B.A., Fraser D.D., Weiss S. (1993). bFGF regulates the proliferative fate of unipotent (neuronal) and bipotent (neuronal/astroglial) EGF-generated CNS progenitor cells. Neuron.

[44] Bottai D., Cigognini D., Madaschi L., Adami R., Nicora E., Menarini M., Di Giulio A.M., Gorio A. (2010). Embryonic stem cells promote motor recovery and affect inflammatory cell infiltration in spinal cord injured mice. Exp. Neurol..

[45] Sharp J., Frame J., Siegenthaler M., Nistor G., Keirstead H.S. (2010). Human embryonic stem cell-derived oligodendrocyte progenitor cell transplants improve recovery after cervical spinal cord injury. Stem Cells.

[46] Hawryluk G.W.J., Mothe A., Wang J., Wang S., Tator C., Fehlings M.G. (2012). An in vivo characterization of trophic factor production following neural precursor cell or bone marrow stromal cell transplantation for spinal cord injury. Stem Cells Dev..

[47] Younsi A., Zheng G., Scherer M., Riemann L., Zhang H., Tail M., Hatami M., Skutella T., Unterberg A., Zweckberger K. (2020). Treadmill training improves survival and differentiation of transplanted neural precursor cells after cervical spinal cord injury. Stem Cell Res..

[48] Hofstetter C.P., Holmström N.A.V., Lilja J.A., Schweinhardt P., Hao J., Spenger C., Wiesenfeld-Hallin Z., Kurpad S.N., Frisén J., Olson L. (2005). Allodynia limits the usefulness of intraspinal neural stem cell grafts; directed differentiation improves outcome. Nat. Neurosci..

[49] Abematsu M., Tsujimura K., Yamano M., Saito M., Kohno K., Kohyama J., Namihira M., Komiya S., Nakashima K. (2010). Neurons derived from transplanted neural stem cells restore disrupted neuronal circuitry in a mouse model of spinal cord injury. J. Clin. Investig..

[50] Nori S., Okada Y., Yasuda A., Tsuji O., Takahashi Y., Kobayashi Y., Fujiyoshi K., Koike M., Uchiyama Y., Ikeda E. (2011). Grafted human-induced pluripotent stem-cell-derived neurospheres promote motor functional recovery after spinal cord injury in mice. Proc. Natl. Acad. Sci. USA.

[51] Butt A.M., Hornby M.F., Kirvell S., Berry M. (1997). Platelet-derived growth factor delays oligodendrocyte differentiation and axonal myelination in vivo in the anterior medullary velum of the developing rat. J. Neurosci. Res..

[52] Hu J.-G., Fu S.-L., Wang Y.-X., Li Y., Jiang X.-Y., Wang X.-F., Qiu M.-S., Lu P.-H., Xu X.-M. (2008). Platelet-derived growth factor-AA mediates oligodendrocyte lineage differentiation through activation of extracellular signal-regulated kinase signaling pathway. Neuroscience.

[53] Stronati E., Conti R., Cacci E., Cardarelli S., Biagioni S., Poiana G. (2019). Extracellular vesicle-induced differentiation of neural stem progenitor cells. Int. J. Mol. Sci..

[54] Rossi S.L., Nistor G., Wyatt T., Yin H.Z., Poole A.J., Weiss J.H., Gardener M.J., Dijkstra S., Fischer D.F., Keirstead H.S. (2010). Histological and functional benefit following transplantation of motor neuron progenitors to the injured rat spinal cord. PLoS ONE.

[55] Ogawa Y., Sawamoto K., Miyata T., Miyao S., Watanabe M., Nakamura M., Bregman B.S., Koike M., Uchiyama Y., Toyama Y. (2002). Transplantation of in vitro-expanded fetal neural progenitor cells results in neurogenesis and functional recovery after spinal cord contusion injury in adult rats. J. Neurosci. Res..

[56] Lee K.-Z., Lane M.A., Dougherty B.J., Mercier L.M., Sandhu M.S., Sanchez J.C., Reier P.J., Fuller D.D. (2014). Intraspinal transplantation and modulation of donor neuron electrophysiological activity. Exp. Neurol..

[57] Younsi A., Zheng G., Scherer M., Riemann L., Zhang H., Tail M., Hatami M., Skutella T., Unterberg A., Zweckberger K. (2020). Three Growth Factors Induce Proliferation and Differentiation of Neural Precursor Cells In Vitro and Support Cell-Transplantation after Spinal Cord Injury In Vivo. Stem Cells Int..

[58] Hernandez M., Patzig J., Mayoral S.R., Costa K.D., Chan J.R., Casaccia P. (2016). Mechanostimulation Promotes Nuclear and Epigenetic Changes in Oligodendrocytes. J. Neurosci..

[59] Hines J.H., Ravanelli A.M., Schwindt R., Scott E.K., Appel B. (2015). Neuronal activity biases axon selection for myelination in vivo. Nat. Neurosci..

[60] Ahuja C.S., Nori S., Tetreault L., Wilson J., Kwon B., Harrop J., Choi D., Fehlings M.G. (2017). Traumatic spinal cord injury—Repair and regeneration. Clin. Neurosurg..

[61] Riemann L., Younsi A., Scherer M., Zheng G., Skutella T., Unterberg A.W., Zweckberger K. (2018). Transplantation of neural precursor cells attenuates chronic immune environment in cervical spinal cord injury. Front. Neurol..

[62] Donnelly D.J., Popovich P.G. (2008). Inflammation and its role in neuroprotection, axonal regeneration and functional recovery after spinal cord injury. Exp. Neurol..

[63] Hamers F.P., Lankhorst A.J., van Laar T.J., Veldhuis W.B., Gispen W.H. (2001). Automated quantitative gait analysis during overground locomotion in the rat: Its application to spinal cord contusion and transection injuries. J. Neurotrauma.

[64] Schaal S.M., Kitay B.M., Cho K.S., Lo T.P., Barakat D.J., Marcillo A.E., Sanchez A.R., Andrade C.M., Pearse D.D. (2007). Schwann cell transplantation improves reticulospinal axon growth and forelimb strength after severe cervical spinal cord contusion. Cell Transplant..

[65] Ferguson A.R., Irvine K.A., Gensel J.C., Nielson J.L., Lin A., Ly J., Segal M.R., Ratan R.R., Bresnahan J.C., Beattie M.S. (2013). Derivation of Multivariate Syndromic Outcome Metrics for Consistent Testing across Multiple Models of Cervical Spinal Cord Injury in Rats. PLoS ONE.

[66] Whishaw I.Q., Pellis S.M., Gorny B., Kolb B., Tetzlaff W. (1993). Proximal and distal impairments in rat forelimb use in reaching follow unilateral pyramidal tract lesions. Behav. Brain Res..

[67] Alluin O., Karimi-Abdolrezaee S., Delivet-Mongrain H., Leblond H., Fehlings M.G., Rossignol S. (2011). Kinematic study of locomotor recovery after spinal cord clip compression injury in rats. J. Neurotrauma.

[68] Stokes B.T., Reier P.J. (1992). Fetal grafts alter chronic behavioral outcome after contusion damage to the adult rat spinal cord. Exp. Neurol..

[69] Behrmann D.L., Bresnahan J.C., Beattie M.S., Shah B.R. (1992). Spinal Cord Injury Produced by Consistent Mechanical Displacement of the Cord in Rats: Behavioral and Histologic Analysis. J. Neurotrauma.

[70] Tashiro S., Nishimura S., Shinozaki M., Takano M., Konomi T., Tsuji O., Nagoshi N., Toyama Y., Liu M., Okano H. (2018). The Amelioration of Pain-Related Behavior in Mice with Chronic Spinal Cord Injury Treated with Neural Stem/Progenitor Cell Transplantation Combined with Treadmill Training. J. Neurotrauma.

[71] Liu M., Li K., Wang Y., Zhao G., Jiang J. (2020). Stem Cells in the Treatment of Neuropathic Pain: Research Progress of Mechanism. Stem Cells Int..

[72] Zweckberger K., Liu Y., Wang J., Forgione N., Fehlings M.G. (2015). Synergetic use of neural precursor cells and self-assembling peptides in experimental cervical spinal cord injury. J. Vis. Exp..

[73] Zhang H., Younsi A., Zheng G., Tail M., Harms A.-K., Roth J., Hatami M., Skutella T., Unterberg A., Zweckberger K. (2021). Sonic Hedgehog modulates the inflammatory response and improves functional recovery after spinal cord injury in a thoracic contusion-compression model. Eur. Spine J..

[74] BASSO D.M., BEATTIE M.S., BRESNAHAN J.C. (1995). A Sensitive and Reliable Locomotor Rating Scale for Open Field Testing in Rats. J. Neurotrauma.

[75] Isu T., Iizuka T., Iwasaki Y., Nagashima M., Akino M., Abe H. (1991). Spinal cord herniation associated with an intradural spinal arachnoid cyst diagnosed by magnetic resonance imaging. Neurosurgery.

[76] Metz G.A.S., Merkler D., Dietz V., Schwab M.E., Fouad K. (2000). Efficient testing of motor function in spinal cord injured rats. Brain Res..

[77] Liu Y., Ye H., Satkunendrarajah K., Yao G.S., Bayon Y., Fehlings M.G. (2013). A self-assembling peptide reduces glial scarring, attenuates post-traumatic inflammation and promotes neurological recovery following spinal cord injury. Acta Biomater..

[78] Watanabe T., Michaelis T., Frahm J. (2001). Mapping of retinal projections in the living rat using high-resolution 3D gradient-echo MRI with Mn2+-induced contrast. Magn. Reson. Med..

[79] Narita K., Kawasaki F., Kita H. (1990). Mn and Mg influxes through Ca channels of motor nerve terminals are prevented by verapamil in frogs. Brain Res..

[80] Sloot W.N., Gramsbergen J.B. (1994). Axonal transport of manganese and its relevance to selective neurotoxicity in the rat basal ganglia. Brain Res..

[81] Pautler R.G., Mongeau R., Jacobs R.E. (2003). In vivo trans-synaptic tract tracing from the murine striatum and amygdala utilizing manganese enhanced MRI (MEMRI). Magn. Reson. Med..

[82] Freitag M.T., Márton G., Pajer K., Hartmann J., Walder N., Rossmann M., Parzer P., Redl H., Nógrádi A., Stieltjes B. (2015). Monitoring of Short-Term Erythropoietin Therapy in Rats with Acute Spinal Cord Injury Using Manganese-Enhanced Magnetic Resonance Imaging. J. Neuroimaging.

[83] Walder N., Petter-Puchner A.H., Brejnikow M., Redl H., Essig M., Stieltjes B. (2008). Manganese enhanced magnetic resonance imaging in a contusion model of spinal cord injury in rats: Correlation with motor function. Investig. Radiol..

[84] Stieltjes B., Klussmann S., Bock M., Umathum R., Mangalathu J., Letellier E., Rittgen W., Edler L., Krammer P.H., Kauczor H.-U. (2006). Manganese-enhanced magnetic resonance imaging for in vivo assessment of damage and functional improvement following spinal cord injury in mice. Magn. Reson. Med..

